# Longterm cognitive dysfunction in paediatric brain tumour survivors - the need for multifactorial risk screening

**DOI:** 10.1192/bjo.2021.174

**Published:** 2021-06-18

**Authors:** Ashy Rengit

**Affiliations:** Kent & Medway NHS Partnership Trust

## Abstract

**Aims:**

Identify common risk factors for longterm cognitive dysfunction in PBTS (paediatric brain tumour survivors) Examine how various paediatric cancer treatment modalities affect cognitive outcomes Consider baseline features which may increase the risk of cognitive dysfunction in PBTS

**Method:**

Current research into the neuropsychiatric sequelae of childhood brain tumours is limited, therefore review of the literature was conducted to identify research within this field.

Databases

Google Scholar - papers accessed via the University of Brighton or Sussex online library

NICE HDAS - HMIC, AMED, MEDLINE, BNI, PsycINFO, CINAHL, Pubmed, EMBASE & EMCARE

Mendeley reference manager - papers for background reading

Search terms

PICO(T) method - Population (Cancer Survivors), Intervention (Cancer Treatment), Comparison (Brain tumour), Outcome (Cognitive dysfunction) & Time (Childhood & adolescence) Boolean operators (AND/OR), truncation and wildcard search functions were also utilised.

Inclusion criteria; no limits on date, study type or gender, however, study results were limited by age - as the research focus was restricted to children and adolescents.

Excluded results; papers which did not meet inclusion criteria, duplicate studies, studies measuring non-cognitive cancer outcomes or investigating non-cortical tumours, non-English language studies with no available English translations.

**Result:**

Common risk factors - certain tumour types (glioneuronal tumours or gliomas) or inner cortical tumour sites e.g. were more vulnerable to epileptogenesis. In particular, seizures which were prolonged and treatment-resistant were associated with a greater degree of cognitive dysfunction.

Impact of various cancer treatment modalities - overall results understandably suggested that patients are more likely to develop cognitive deficits following brain tumour treatment. In particular, partial tumour resection (especially if epileptogenic), whole-brain irradiation, cranial radiotherapy and chemotherapy were more likely to impact cognitive function.

Baseline features that may increase likelihood of cognitive dysfunction e.g. intellectual disability or education level were not noted in the reviewed literature.

**Conclusion:**

Cancer is one of the leading causes of global child mortality, and younger populations often present to paediatric oncology services with brain tumour involvement. Current childhood brain tumour research has begun to recognise that many young survivors develop into adulthood with cognitive sequelae impacting quality of life measures. However, existing evidence is also limited and requires further research to produce a standardised clinical tool for screening various risk factors which may increase longterm risk of cognitive dysfunction and subsequent difficulties with daily life.

